# Management of forearm nonunions: current concepts

**DOI:** 10.1007/s11751-011-0125-0

**Published:** 2011-11-24

**Authors:** Peter Kloen, Geert A. Buijze, David Ring

**Affiliations:** 1 Department of Orthopaedic Surgery, Academic Medical Center, Meibergdreef 9, 1100 DD Amsterdam, The Netherlands; 2 Department of Orthopaedic Surgery, Massachusetts General Hospital, Boston, MA USA

**Keywords:** Nonunion, Forearm, Ulna, Radius, Compression plate, Internal fixation

## Abstract

Forearm nonunions are uncommon but severely disabling and challenging to treat. Multiple factors have been associated with the establishment of forearm nonunions such as fracture location and complexity, patient characteristics and surgical technique. Treatment of diaphyseal forearm nonunions differs from that of other type of diaphyseal nonunions because of the intimate relationship between the radius and ulna and their reciprocal movement. There is a wide variation of surgical techniques, and the optimal choice of management remains subject to debate. In this review, we aim to summarize the available evidence in the literature on forearm nonunions and combine it with practical recommendations based on our clinical experience to help guide the management of this complex problem*.*

## Introduction

Modern plate-and-screw fixation most notably the 4.5 mm dynamic compression plates (DCP) developed by the AO- essentially “solved” the problem of diaphyseal forearm fractures [1]. Malunion and nonunion, once frequent, are now uncommon, and the short- and long-term impairment and disability are limited. Nonunion is associated with technical shortcomings (a plate that is too short or too weak) or injury severity (bone loss, poor soft tissue cover, infection or contamination) [2].

Most forearm nonunions are atrophic and many have an associated bony defect. The variations in treatment relate primarily to how defects are handled. The options include autogenous cancellous bone graft, autogenous corticancellous bone graft and vascularized bone grafts (typically for the radius).

## Nonunions of the olecranon and proximal ulna

Nonunion after operative treatment of a displaced fracture of the olecranon is very uncommon and usually related to patient noncompliance and/or insufficient surgical technique with implant failure. Nonunions of the proximal ulna are associated with complex injury patterns such as anterior or posterior fracture-dislocations of the olecranon and posterior Monteggia fractures. In particular, posterior Monteggia injuries in adults typically occur in older women with poor bone quality. They can be difficult to secure resulting in inadequate plate fixation and subsequent nonununion.

**Fig. 1:**
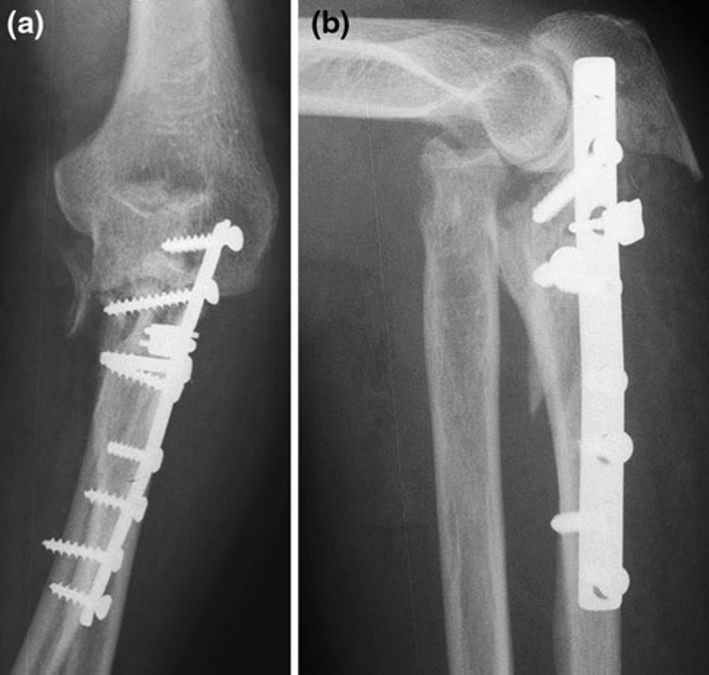
AP and lateral radiograph showing a nonunion after plate fixation of a Monteggia fracture. The plate is not on the tension (=dorsal) side but rather on the medial side. The apex posterior deformity recurred with subluxation of the radial head

In our recent review of failed fixation and nonunion of posterior Monteggia fractures [3], the plate was often positioned medially or laterally—rather than posterior—with only two or three screws in the proximal metaphyseal fragment. When the proximal screws loosened, the apex posterior deformity with subluxation of the radial head from the radiocapitellar joint recurred (Fig. 1). Radial head fracture is part of this injury and often warrants repair or replacement. In patients with ulnohumeral instability reattachment of the lateral collateral ligament may be helpful [3, 4].

**Fig. 2:**
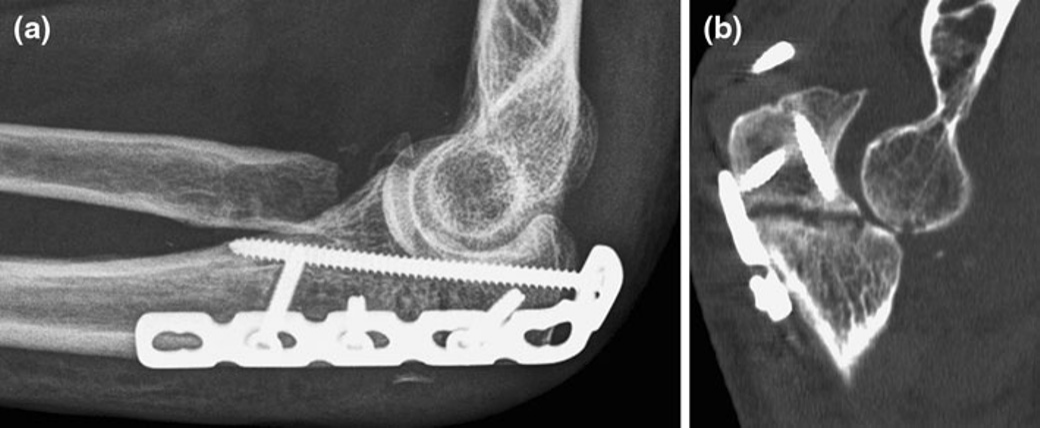
**a** Plain film could not clearly explain symptoms of pain and limited motion after radial head resection and plating of a Monteggia injury; **b** The CT-scan provided much more insight showing a nonunion of the proximal ulna and subluxation of the ulno-humeral joint. Case courtesy Peter Kloen (*Source*: Marti and Kloen [26])

Preoperative evaluation should consist of a detailed neurovascular exam, locating previous incisions and measuring the range of elbow and wrist motion. Specific blood tests are only needed in case of suspected infection (e.g., complete blood count including differential, erythrocyte sedimentation rate and C-reactive protein). The surgeons should note whether the nonunion is atrophic, oligotrophic, or hypertrophic. Previous surgical reports should be scrutinized for detail regarding the type of hardware used, intra-operative difficulties encountered. Posteroanterior and lateral radiographs are required and, in addition, regular 2D and 3D CT can provide useful additional details such as the size and instability or incongruence of the proximal radioulnar joint (Fig. 2).

**Fig. 3:**
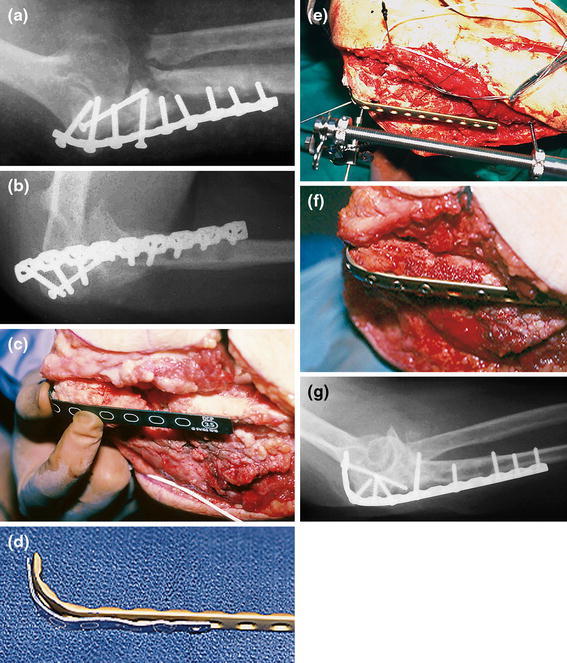
Nonunion after internal fixation of a posterior Monteggia fracture dislocation. **a**, **b** Loosening of the plate and screws and instability; **c**, **d** After hardware removal, debridement, a posterior plate is contoured to cradle the proximal ulna; **e** Using the femoral distractor alignment was obtained; **f** Final appearance intra-operatively after plating and bone grafting; **g** Postoperative radiograph. Case courtesy Jesse B. Jupiter (*Source*: Marti and Kloen [26]) (Copyright owned by David Ring, MD, PhD)

**Fig. 4:**
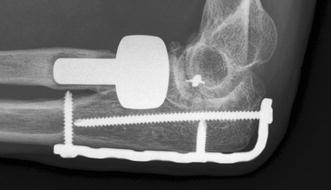
If good bone contact is present, compression of the nonunion and orthogonal screw position will suffice (patient also shown in Fig. 2)

During the surgical procedure, previous failed hardware is removed followed by debridement of the synovial membranes and inflammatory tissue around the nonunion. The nonunion can be mobilized and opened using a laminar spreader. After thorough debridement using drills, curettes and rongeurs, the ulna is temporary stabilized with K-wires. It is important to position the 3.5 mm LC-DCP (low contact-dynamic compression plate) or LCP (locking compression plate) on the crista (or dorsal aspect) of the proximal ulna because that is the tension side. The crista of the ulna should first be cleared by sweeping the m. extensor carpi ulnaris toward dorsal (lateral) and the m. flexor carpi ulnaris toward volar (medial) for a few millimeters using a small periosteal elevator or knife. The plate is contoured to wrap around the olecranon, allowing for more screws to be placed in the proximal fragment (Fig. 3). If proximal screws are placed orthogonal to the more distal screws a rigid interlocking construct can be achieved with relatively few screws provided there is good bone contact and compression (Fig. 4) [5–7]. If the proximal fragment is too small for rigid screw purchase, it might better be excised followed by meticulous reattachment of the m. triceps tendon. A kinematic study showed that up to 6 mm of posteromedial olecranon resection does not lead to clinical symptoms of valgus angulation [8]. In elderly patients with severe arthritis or osteoporosis total elbow prosthesis can be used as salvage.

## Nonunions of the radial head and neck

Nonunions of the radial head are uncommon [9]. After operative treatment they are associated with complex fractures with more than three fragments [2]. The repaired radial head may function as a spacer, but the implant failure is usually symptomatic enough to benefit from radial head excision. By that time the ligaments are usually healed and the radial head needs not be replaced.

**Fig. 5:**
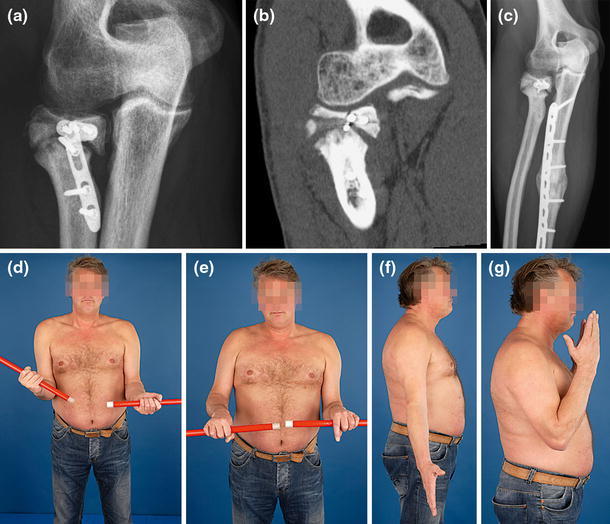
A persistent nonunion of the radial neck can be relatively asymptomatic. **a**–**c** Patient sustained an ulna shaft refracture after plate ulna removal from a previous Monteggia injury. He chose refixation of the ulna and only partial removal of the radial fixation; **d**–**g**: He has no complaints and almost full range of motion. Case courtesy Peter Kloen (*Source*: Marti and Kloen [26])

**Fig. 6:**
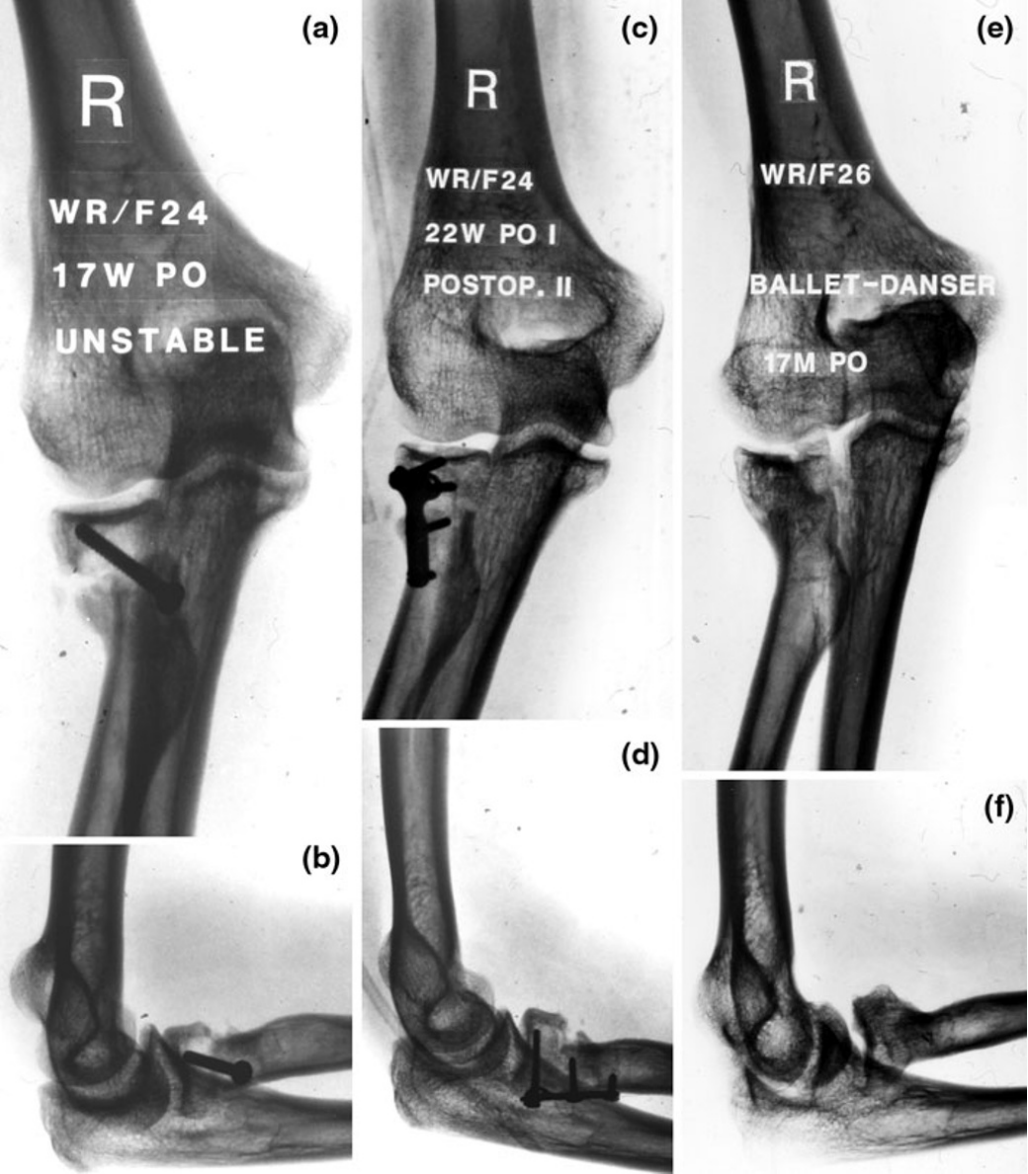
Nonunion of the radial neck treated with revision ORIF (2.7 mm T-plate) and autologous bone grafting. Case courtesy Rene K. Marti (*Source*: Marti and Kloen [26])

Nonunions of the radial neck after nonoperative treatment may occur more often than diagnosed, because they are associated with few symptoms and excellent elbow function (Fig. 5) [9, 10]. Both operative and nonoperative (radiographic) nonunions diagnosed more than a year after injury have been noted to heal eventually, so in the absence of other indications, radiographic appearance is not an indication for intervention. If symptomatic, surgical revision with impacted bone graft and plate fixation is technically demanding but possible (Fig. 6). Interestingly, a recent study by Neumann et al. [11] suggested that fixation of a reconstructed radial head to the radial shaft is not always necessary.

In their report, there was no difference in clinical outcome between patients with a Mason III radial head fracture that had fixation to the radial neck versus those where no fixation was performed.

## Diaphyseal forearm nonunions

**Fig. 7:**
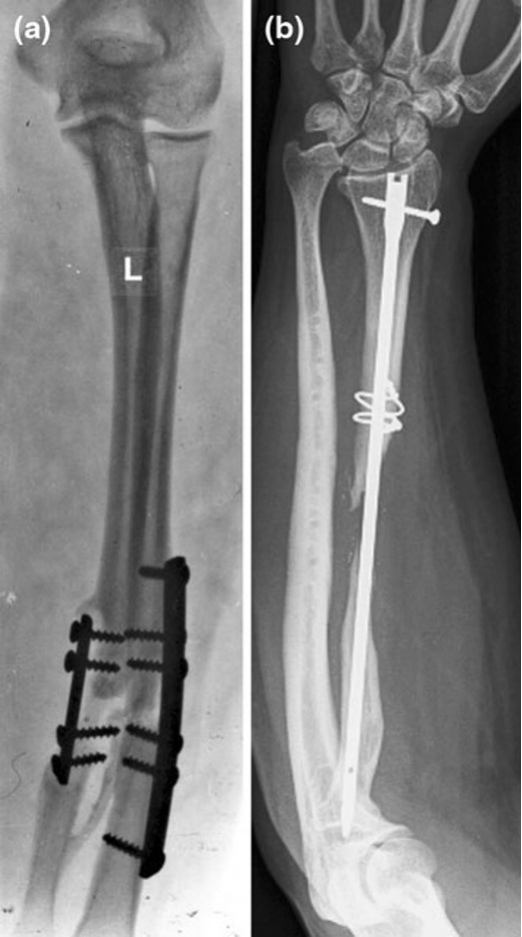
Technical errors in fixation of fracture and nonunion. **a** Plate too short; **b** Lack of rotational control and “biology” by IM fixation without bone graft of an atrophic radial shaft nonunion

Modern compression plate-and-screw fixation has proven to be a relatively straightforward procedure in adults. Low complication rates and nonunion rates below 5% have been reported in large series [1, 12, 13]. Despite these advances in treatment and outcome, controversies still exist regarding bone grafting for acute fractures, type and length of the plate and the risk of refractor after plate removal [13, 14]. Diaphyseal forearm nonunions are rare but severely disabling as dysfunction extends to the elbow and wrist, which limits the ability to place the hand in space [2, 15]. Risk factors include comminution, high-energy fractures, open fractures and technical shortcomings of surgery (Fig. 7). Causes are usually multifactorial.

Preoperative evaluation is as described above except for that there is no additional role for CT. It is important to know whether the nonunion is atrophic, oligotrophic, or hypertrophic as it will determine the surgical strategy. In general, the strategy is to adhere to “biologic surgical technique” with preservation of soft tissue attachments.

**Fig. 8:**
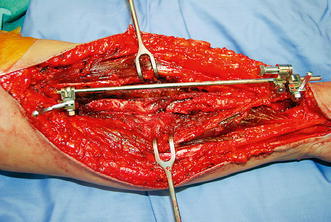
Wide exposure and debridement of a radial shaft nonunion. The intraoperative ex-fix helps alignment and obtaining length

**Fig. 9:**
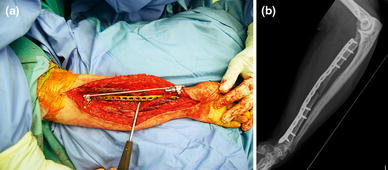
**a** A bridging plate and autologous cancellous bone grafting for an atrophic radial shaft nonunion; **b** Consolidation—with slow remodeling—was seen after 1 year. She had no symptoms and near full ROM

Atrophic and oligotrophic nonunions require debridement of interposed fibrous tissue and necrotic and devitalized areas. Opening the sclerotic bone ends and roughening the fracture surface stimulates bleeding and subsequent healing response. The medullar canal is opened on both ends of the nonunion using a 2 mm drill and on either side, the bone is decorticated using a sharp osteotome over a length of about 2 cm. The soft tissues and periosteum are not to be separated from the bony petals. In case of an ulna and radial nonunion with a small defect, the bone can be shortened symmetrically. However, it is preferable to maintain length using bridge plating and grafting. In large defects of the radius, there often is an ulna positive variance with a concomitant disruption in the distal radioulnar joint (DRUJ). Soft tissue contracture can complicate restoration of length. In such cases, a combination of release and intra-operative distraction can be performed by using an AO distractor (or external fixator for large defects) (Fig. 8). For smaller defects, the articulated tension device can be used in distraction mode or a laminar spreader can be applied between the plate and a separate cortical screw. During distraction, the median nerve may be jeopardized and should be constantly visualized. Once adequate length is obtained, a premolded plate is applied in bridging fashion (Fig. 9). The use of 3.5 mm plates (DCP, LC-DCP, LCP) is preferred over 4.5 mm plates (too bulky in the forearm) and intramedullary wires, K-wires, simple lag screws or 1/3 tubular plates (too unstable) [7, 13]. We advise not to use intramedullary nailing as there is lack of compression and rotational control (Fig. 7b) [13]. Our data suggest that standard (nonlocking) plate-and-screw fixation can have a high success rate even in osteoporotic bone. Locking fixation can be used as well but has not been proven superior [16].

**Fig. 10:**
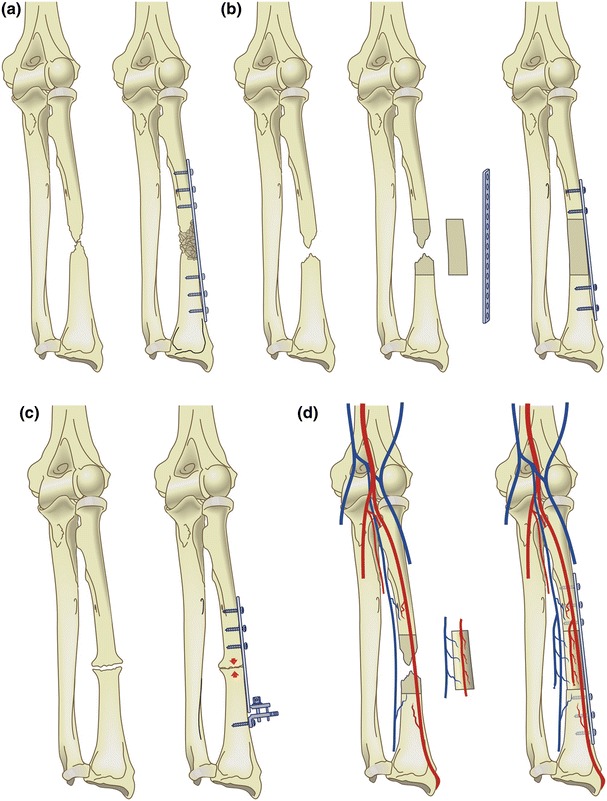
Bone grafting for diaphyseal forearm nonunion. **a** For atrophic nonunion, we prefer autologous cancellous bone graft for defects up to 6 cm; **b** Others have used autologous nonvascularized bone blocks; **c** hypertrophic nonunion only need compression; **d** A vascularized bone grafter (or osteoseptocutaneous flap) requires microsurgical expertize with donor-site morbidity

The choice of bone graft is ongoing topic of debate (Fig. 10) [2, 13–15]. For defects up to 6 cm after reconstruction, our preference (and that of others) is to use autologous cancellous bone graft for an atrophic or oligotrophic nonunion (Fig. 10a) [13, 15]. It is important to petal both sides (1.5–2 cm) of the nonunion and to open the medullar canal to remove the sclerotic cap using a drill. We generally harvest the graft from the inside of the anterior iliac crest. Donor-site deformity and morbidity can be avoided if done appropriately. Vascularization of a corticocancellous graft occurs within a few weeks if the soft tissue envelope is compliant and well-vascularized. Other authors preferred the use of nonvascularized bone blocks, though some of them protected the repair postoperatively in a cast for a long period (Fig. 10b) [17, 18]. For defects between 6 and 10.5 cm, there the choice of bone graft is more controversial [19]. In more complex cases of a substantial bone defect with concomitant poor-vascularized soft tissue, the use of an osseocutaneous-free flap is a viable alternative (Fig. 10d). However, this requires microsurgical expertize but it can create a suitable soft tissue environment in addition to bony continuity [20]. We do not advise a free fibular transfer, despite its high success rate, as there are disadvantages including donor-site morbidity, the need for microsurgical expertize and a higher risk of infection (Fig. 9).

**Fig. 11:**
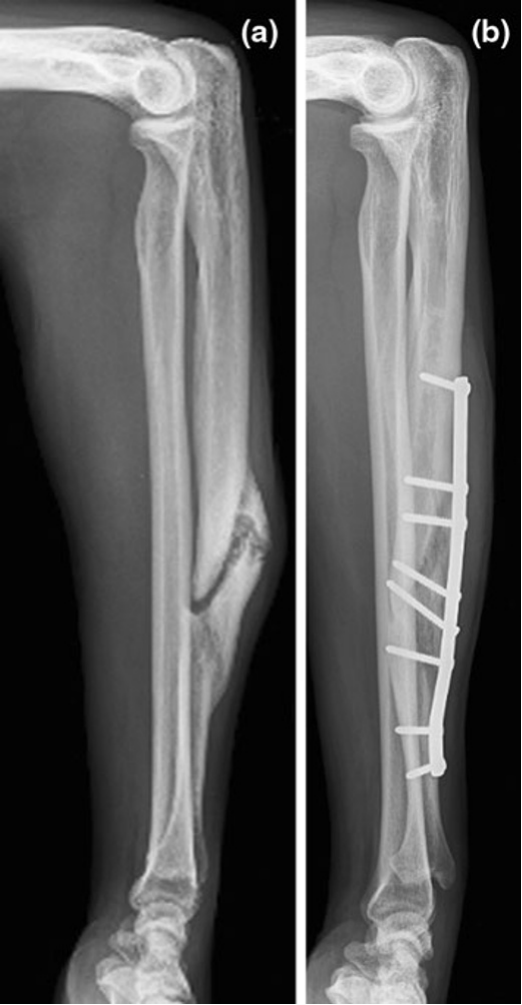
**a**, **b** Ideally use standard AO-techniques using compression, lag screws and relatively high plate/screw ratio

In hypertrophic nonunion, there is no need for bone grafting (Fig. 10c). By removing some of the callus, the plate can be positioned better. We advise the use of long 3.5 mm plates (DCP, LC-DCP, LCP) with a high plate/screw ratio and an additional lag screw if possible (Fig. 11).

## Nonunions of the distal radius

Nonunions of a distal radius fracture are extremely uncommon (approximately 0.2%) [21–23]. Risk factors include low-energy fractures, impaction, metaphyseal fractures, concomitant fractures of the distal ulna shaft and concomitant DRUJ lesion combined with a medical condition compromising bone healing. Nonunion should be expected if patients present with a painful progressive cosmetic deformity of the wrist resulting in decreased hand function. Although the necessity for operative treatment is undisputed, there is no consensus on the optimal technique. Options range from formal ORIF with or without resection of the distal ulna to wrist arthrodesis. Formal ORIF can be successful even in nonunions with less than 5 mm of subchondral bone, although more postoperative complications were seen in patients with smaller distal fragments [21]. Improved implants have facilitated fixation. Wrist arthrodesis should only be considered as a salvage procedure [21].

**Fig. 12:**
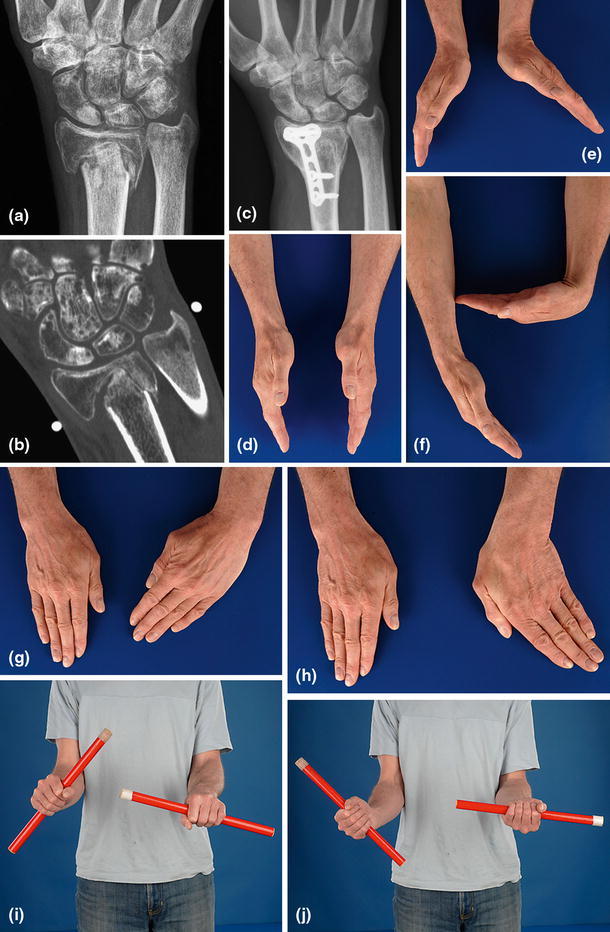
**a**, **b** Plain AP radiograph and CT of a distal radius nonunion. Detail proved by CT facilitates pre-operative planning; **c** Placement of a tricortical graft allow for some correction of radial length. Intrinsic stability provided by the soft tissue tensioning increased stability in the nonlocking era; **d**–**j** Wrist and forearm function at 7 years follow up

**Fig. 13:**
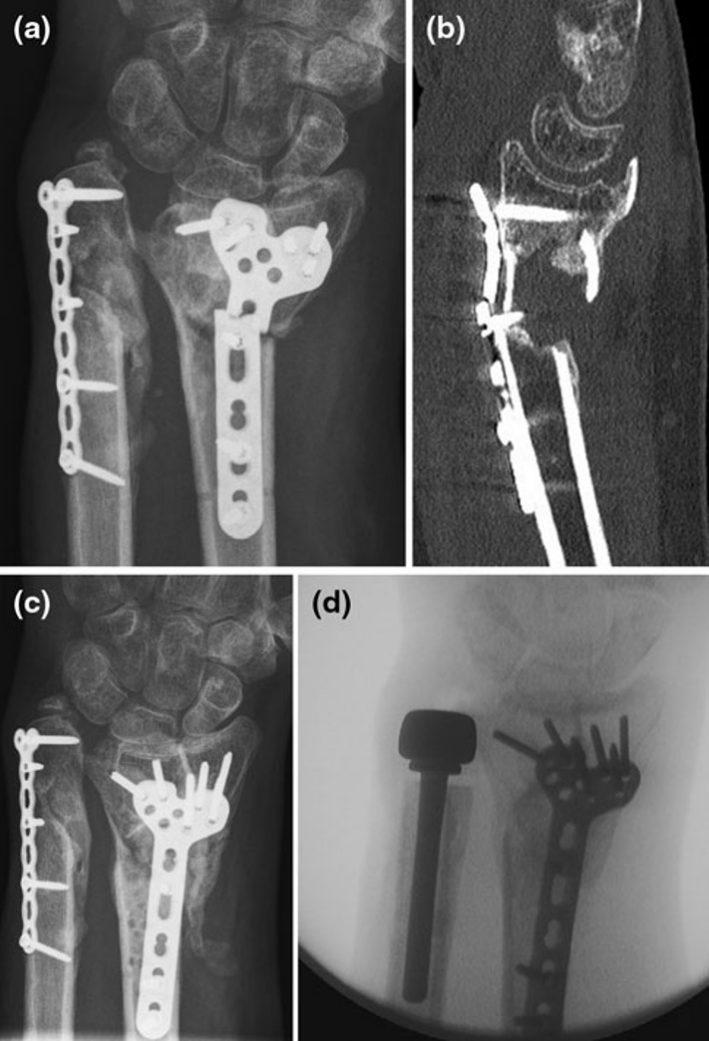
**a**, **b** A nonunion after a Gustillo Grade 2 open complex distal ulna and radius fracture was treated with revision ORIF and bone graft of the distal radius (**c**). The ulna plus deformity was later salvaged with ulna head prosthesis by a plastic surgeon (**d**)

Preoperative planning is as described above. Two- or three-dimensional CT can be of additional value in determining the size and instability or incongruence of the DRUJ (Fig. 12). Surgery aims primarily at debriding the nonunion by removing all fibrous and synovial interposed tissues. After removing the sclerotic endcaps, the intramedullary canal is opened on both sides with a small drill. A small distractor can be helpful to reduce the fragments. Deformity in the sagittal and coronal planes can be corrected by an opening wedge. A radial deviation deformity can be corrected in part by lengthening the m. brachioradialis and m. flexor carpi radialis tendon [23]. The use of orthogonal plates allows for more points of fixation in case of small distal fragments [23]. Angular stable fixation is more secure in these small and often osteoporotic fragments. A cancellous bone graft can be used because of the fixed angle fixation stability. A tricortical opening wedge will provide intrinsic stability because of the tightening of the soft tissue (Fig. 12c). If there is severe shortening of the distal radius that cannot be corrected, resection of the distal ulna (Darrach procedure) of nowadays preferably placement of an ulna head prosthesis is available (Fig. 13).

## Infected nonunions of the forearm

**Fig. 14:**
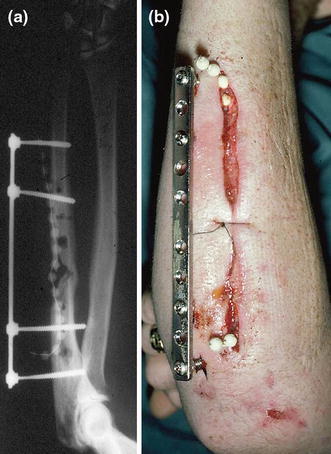
**a**, **b** A modified DCP was used to stabilize and infected ulna nonunion. Case courtesy Chris van der Werken (*Source*: Marti and Kloen [26])

For these complex cases, aggressive debridement, removal of hardware, temporary external fixation and antibiotic treatments are advised. Reconstruction should be planned only when infection has subsided based on clinical and laboratory parameters. In cases of extensive scarring and devitalization of soft tissues, an osteocutaneous fibular graft with an anastomosis to the radial or ulnar vessels is a viable option. Recently, a large series of treatment of infected diaphyseal forearm nonunions was reported. The authors used a combination of aggressive debridement, definitive fixation after 7–14 days, bone grafting for segmental defects, leaving wounds open by secondary intention, using intravenous antibiotics and early mobilization. Their satisfactory results suggest an alternative to temporary external fixation [24]. A modified DCP or LCP can be used as an external fixator can be used for infected ulna nonunion (Fig. 14).

## Postoperative management

For proximal and midshaft nonunions, an above-elbow splint (well-padded dorsally) allowing for wound healing is given for 7–10 days. For distal radius nonunions, a below-elbow splint suffices. Rehabilitation is dependent on the surgeon’s estimate of the achieved fixation stability. Patients with atrophic and oligotrophic nonunions should refrain from pro-/supination as well as any lifting for a minimum of 6 weeks. Patients with hypertrophic nonunions in which a rigid compression plating has been achieved generally do well with early mobilization. Swelling and stiffness can be minimized by elevation of the hand and active hand exercises. Active assisted elbow, forearm and wrist exercises can be initiated as comfort allows. Passive manipulation by a physical therapist is not allowed. Once early consolidation is established on radiographs, it is reliable to start resistive exercises for strengthening. This may take much longer in large defects treated with autologous bone grafting in a healthy environment.

Hardware removal is not routinely removed because of the known risk for refracture. In fact, many of the patients in our series presented with a refracture after plate removal. For proximal ulna nonunions treated with dorsal plating, exceptions are made, as the subcutaneous position makes the plate prominent and bothersome. In any case, hardware should not be removed within 18 months after healing. Healing on radiographs is determined by incorporation of the graft (if used), crossing trabeculae and full remodeling. Volar plate fixation of the distal radius rarely results in hardware prominence and the need for removal.

## Authors’ recommendations

The authors see no role for minimally invasive techniques as limited exposure will likely compromise the ability to obtain anatomic alignment. Stability of fixation is important in achieving early consolidation. Shortening through compression might lead to abnormalities at the wrist (creating an ulna minus or ulna plus), needing a secondary shortening or lengthening operation. The fixation of choice is a relatively long 3.5-mm compression plate. Most authors advise 6 cortices on each side of the fracture.

Longer plates (3.5 mm) with a high plate-span/screw ratio are preferred. Realignment of the DRUJ is assessed with fluoroscopy and passive forearm rotation. In case of degenerative changes of the DRUJ or persistent incongruity of the joint, prosthetic replacement of the distal ulna currently has preference over distal ulna resection.

## Outcome

Due to its rarity there is a paucity of data on mid-term and long-term functional outcome for operative treatment of forearm nonunions (in particular on patient-based outcome) as surgeons tend to focus primarily on achieving union. The few retrospective studies reporting on functional outcome used the system of Anderson et al. [1] which strictly reflects the range of elbow, forearm and wrist motion. It rates an united fracture with <10° loss of elbow or wrist motion and <25% loss of forearm rotation as excellent, a healed fracture with <20° loss of elbow or wrist motion and <50% loss of forearm rotation as satisfactory, a healed fracture with more than 30° loss of elbow or wrist motion and more than 50% loss of forearm rotation as unsatisfactory, and a malunion, persistent nonunion or unresolved chronic osteomyelitis as failure. Outcomes based on this system widely vary across the few cohorts who may reflect the heterogeneity of the injury complexity [2, 13, 15, 25].

Ring et al. reported on the functional outcome of 35 patients with an atrophic diaphyseal forearm nonunion treated with 3.5-mm plate-and-screw fixation and autogenous cancellous bone grafting [2]. At a minimum of 1 year follow-up, they noted substantial functional improvement in all their patients. According to the Anderson classification (after an average of 43 months), 5 patients (14%) had an excellent result, 18 (51%) had a satisfactory result, 11 (31%) had an unsatisfactory result (because of elbow stiffness related to associated elbow injuries in three and because of wrist stiffness in eight) and 1 (3%) had a poor result (because of malunion). They found that the functional results were diminished by residual stiffness related to the original trauma, previous operations, and prolonged immobilization and disuse of the limb.

Faldini et al. reported on two cohorts of forearm nonunions: the first cohort of 20 patients treated with compression plating and autogenous fibular bone grafting [15] and the second cohort of 14 patients treated with compression plating and allografts [25]. In the first cohort (minimum of 12 years follow-up), 8 patients (40%) had excellent results, 10 (50%) had satisfactory results, 2 (10%) had unsatisfactory results and none had poor results or failures according to the Anderson scoring system [15]. The mean VAS for pain was 1 (range, 0–3). Patients resumed activities of daily living (ADL) at 2 months after surgery, original work activity at 3–4 months after surgery and sports activities at 4–5 months after surgery. Grip strength was normal in 8 patients, slightly limited in 11 patients and severely limited in 1. The results of their second cohort (minimum of 2 years follow-up) were markedly similar to the first cohort [25].

We recently retrospectively reviewed a cohort of 47 patients with 51 diaphyseal forearm nonunions in adults treated with various techniques at our institution during a period of 33 years [13]. According to the Anderson classification (at a minimum of 1 year and average of 6 years), 29 patients (62%) had an excellent result, 8 (17%) had a satisfactory result and 10 (21%) had an unsatisfactory result. No treatments resulted in failure but complications were seen in six patients (13%). The reasons for the unsatisfactory results were limited range of motion of the wrist in 8 patients, elbow stiffness in 1 and a median nerve lesion in 1. The 18 patients that had an open fracture at the time of injury had slightly worse functional results.

In comparison with healed proximal ulna and distal radius nonunions, diaphyseal forearm nonunions generally tend to result in somewhat better functional outcome, likely because the relationships in the proximal and/or DRUJ are not (or less) affected [4, 21, 22].

## Summary

Forearm nonunions are uncommon but challenging. Operative treatment with adequate debridement, eradication of infection and stable fixation using compression (using lag screws, eccentric drilling and/or AO tensioner device) will lead to high predictable rates of healing. For the diaphyseal nonunion, longer plates with a high plate-span/screw ratio are preferred to achieve a more stable fixation. Segmental defects up to 6 cm can be successfully reconstructed with autogenous corticocancellous bone grafts while larger defects may require free tissue transfer. For the distal radius nonunions, placement of an opening wedge will not only correct deformity but also provide intrinsic stability. Locking plate technology has facilitated fixation of the small distal radius nonunion fragments.

Study results suggest that when following adequate techniques, the vast majority of patients with forearm nonunions can be brought to union and obtain a satisfactory long-term functional outcome.
